# miR-223-5p targeting ERG inhibits prostate cancer cell proliferation and migration

**DOI:** 10.7150/jca.44441

**Published:** 2020-05-18

**Authors:** Yongbao Wei, Junming Peng, Shuyun He, Haijian Huang, Le Lin, Qingguo Zhu, Liefu Ye, Tao Li, Xing Zhang, Yunliang Gao, Xiaochun Zheng

**Affiliations:** 1 Shengli Clinical Medical College of Fujian Medical University, Fuzhou 350001, China; 2 Department of Urology, Fujian Provincial Hospital, Fuzhou 350001, China; 3 Department of Urology, Shenzhen People's Hospital, Second Clinic Medical College of Jinan University, The First Affiliated Hospital of Southern University of Science and Technology, Shenzhen 518020, P.R. China.; 4 Department of Urology, the Second Xiangya Hospital, Central South University, No139. Renmin Road, Changsha 410011, China; 5 Department of Urology, The People's Hospital of Xiangtan Country, Xiangtan, China; 6 Department of Pathology, Fujian Provincial Hospital, Fuzhou 350001, China; 7 Department of Urology, the Traditional Chinese Medicine Hospital of Yangzhou, Yangzhou University of Traditional Chinese Medicine, Yangzhou, Jiangsu 225002, China; 8 Department of Anesthesiology, Fujian Provincial Hospital, Fuzhou 350001, China.

**Keywords:** miR-223-5p, prostate cancer, ERG, biological behavior

## Abstract

Ectopic expression of miR-223-5p, the lagging strand of miR-223 duplex, has been reported acting as anti-tumor miRNA in many cancers. How miR-223-5p influencing prostate cancer (PCa) remains obscure and worth of experimental investigation. In this study, the expressions of miR-223-5p and ERG in common PCa cell lines were detected and compared to RWPE-1, respectively. Then luciferase reporter assay was performed to verify whether miR-223-5p could specifically target and regulate ERG. Further discovery ERG's role in the PCa oncogenesis was also conducted by up or down regulating miR-223-3p expression. We found miR-223-5p was significantly down-regulated in DU145, while it was only up-regulated in LNCaP. Similarly, ERG expression remarkably decreased in both PC-3 and DU145 than that in RWPE-1, but significantly increasing in LNCaP. Luciferase assay demonstrated slightly decreased ERG expression after miR-223-5p-mimics but significantly increased ERG expression after miR-223-5p-inhibtor. Using gene interference, we further confirmed that both ERG mRNA and protein expressions were decreased in all PCa lines transfected ERG siRNA, but increasing in both DU145 and LNCaP cells with miR-223-5p antisense oligonucleotides. MTT assay, Transwell invasion and migration assay supported the function of ERG in PCa oncogenesis. We revealed tumor suppressive abilities of miR-223-5p in PCa by negatively targeting ERG gene. It could serve as a fundamental supplement and extension of our previous study about miR-223-3p in PCa, revealing the coordinative regulation between miR-223-5p and miR-223-3p in PCa cell biological behaviors. Exploration of miR-233-duplex orientated pathway networks may help us develop novel potential therapeutic options for PCa.

## Introduction

Prostate cancer (PCa) is a commonly urogenital solid malignancies, contributing to one of commonest cancer-associated death in aged males worldwide. In 2018, it is projected to be nearly 165 000 cases in the US and may account for nearly 30 000 deaths 1. Despite the seemingly large number of men with this lethal disease, there remain men who are undiagnosed, asymptomatic or with indolent PCa. Particularly, these advanced PCa patients have shorter survival times and poorer quality of life because of metastasis-related symptoms as well as therapy-associated adverse effects. Given that, it is paramount importance to understand the biologic underpinnings of PCa and identify biomarkers for clinical outcomes prediction.

MicroRNAs (miRNAs) are a growing class of small non-coding RNAs; they can influence the post-transcriptional regulation of target genes and thus participate in a variety of biological functions in living organisms 2. Double-strand miRNA contains the guide and lagging strands, while the outcome of lagging strand commonly results in deterioration without regulation of target gene expression. Recent emerging evidences support a novel concept that the passenger strand involves in the cancer pathogenesis. In particular, miR-223-5p as the aberrantly expressed lagging strand, has been reported in few solid malignant neoplasms including vulvar carcinoma 3, non-small cell lung cancer 4 and bladder cancer 5. miR-223-5p could act as anti-tumor miRNA by targeting several oncogenic genes such as E2F8 and ANLN. However, its role in PCa remains unknown and is worth exploring clearly including relevant oncogenic networks.

The ETS-related gene (ERG), as a member of the transcription factor ETS family, has a highly conserved DNA binding domain. ERG plays an important role in the development of multiple systems or organs in the human body, such as angiogenesis and hematopoiesis etc. 6. Of particular interest is that in PCa patients, the TMPRSS2-ERG gene fusion found to occur in about a half of them 7. The fusion gene could lead to a higher expression of ERG, which may play a critical role in the multiple aspects of cell growth and development 8. It is widely believed that ERG is an important driver of epithelial neoplasia in the prostate epithelium and promotes cancer development; its overexpression is also the most consistent among many PCa oncogenes^6^. However, to date there are no clear known mechanisms underlying its oncogenic function in PCa. To fill this gap, we explored whether the mir-223-5p play a crucial role in PCa pathophysiology through interaction, and meanwhile to prove whether its function is achieved by negatively targeting ERG gene. This study may provide new opportunities to elucidate the mechanisms of PCa tumorigenesis.

## Methods

### Cell culture and transfection

We purchased cells of RWPE-1, DU145, PC3 and LNCaP from Cell Bank of Chinese Academy of Sciences (Shanghai, China). We cultured DU145 and LNCaP in RPMI-1640 (Hyclone, USA) as recommended, while PC-3 was in F-12K (Life technologies, USA); both of above media supplemented with 10% FBS. RWPE-1 was grown in prostate epithelial cell medium (PEpiCM, ScienCell, USA). All cell lines were cultured in an incubator with a temperature of 37°C and CO_2_ concentration of 5%. We performed cell transfections by using Lipofectamine™ 2000 complexes (Invitrogen, CA, USA) according to the reference manual. The cell incubation time was 24 or 48 hours. Then the number of cells would be measured under light and fluorescence microscopy.

### RT-qPCR analysis of mRNA

We obtained total miRNA from cells by using Trizol reagent (Invitrogen, USA) according to its illustration. RT-qPCR was carried out on a Pikoreal 96 PCR system (Thermo Fisher Scientific, USA). UltraSYBR Mixture (with ROX) (CWBio, China) and the miRNA Real-Time PCR Assay kit (CWBio, China.) were used to detect and measure relative expression of mRNA as well as miRNA, respectively. We normalized the results and analyzed the data based on the 2^-ΔΔCt^ method. The primers in this study were indicated in Table 1.

### Western blot

We lysed and extracted protein samples 24 h or 48 h after cell transfection. SDS-PAGE gel (Sigma, USA) was prepared and was added into protein sample. Then the protein was heated for 3-5 minutes in a boiling water at 100 ° C to been fully denatured. After cooling to room temperature, the protein sample was directly loaded into the SDS-PAGE gel well. The protein sample was then transferred to a nitrocellulose blotting membrane (PALL, USA). After blocking, it was incubated with ERG antibody (1:200 Proteintech, #14356-1-AP, USA). After overnight at 4 ° C, goat anti-rabbit IgG / HRP (1:6000) was incubated. Then the protein was detected and the relative expression of the protein was read using β-actin as a reference.

### Cell proliferation assay

We detected cell proliferation rate using MTT assay (3-(4,5-dimethylthiazol-2-yl)-2,5-diphenyl tetrazolium bromide, Sigma, USA). We prepared and adjusted a single cell suspension, and then cells were inoculated into 96-well plates with each plate having 1 x 104 cells and each volume was 100 ul. After cultured at 37 ° C in a 5% CO 2 incubator, the cells were transfected with miR-NC or miR-223-5p antisense oligonucleotides respectively and then incubated for 48 hours. Then cells were cultured for 4 h after adding 50 ul of MTT solution into per well. We remove the culture supernatant and dropped 150 ul of dimethyl sulfoxide (DMSO, Sigma, USA) into each well. Then the plate was kept shaking for 10 minutes to allow crystalloid dissolved completely. Following this, at 490 nm, we measured the absorbance of each well under a microplate reader (Huisong MB-530, China) and drawn curve of cell proliferation.

### Transwell chamber for invasion and migration assay

Cell invasion assay was performed as previously 9. Low serum DMEM medium and 20% FBS-DMEM medium were prepared for the upper and lower transwell chambers (Corning, USA), respectively. Matrigel gel (BD, USA) was prepared and was then diluted with serum-free cold cell culture medium DMEM. 100 ul of dilute gel was added to the 24-well chamber. We incubated the transwell at 37 ° C for at least 4-5 h. The gel was lightly washed with serum-free medium. After washing with the medium, the cells were resuspended to obtain a cell concentration of 5 x 105 cells/ml with 1% FBS. Add 200 ul of cell suspension to the upper chamber. 600 ul of cell culture medium containing 5 ug/ml fibronectin as an adhesion subfamily was added to the lower chamber. then cells were incubated at 37 ° C for 20 to 24 h. Residual cells in the upper chamber were wiped off. Remove the transwells, 500 μl of 0.1% crystal violet was added to the plate and the chambers were kept under the crystal violet at 37 °C for 30 min. Then wash the chambers with PBS. Photographing and counting were then performed.

The steps of migration assay were similar, except the upper film not covering Matrigel before the cells added.

### Luciferase reporter assay

Luciferase assay was performed according to previous report 9. Brief, several microRNA targets gene prediction systems website were searched to find target genes of miR-223-5p (PicTar, microRNA, MiRBase and TargetSpy). We found that the 3′UTR of ERG mRNA fragment contained miR-223-5p responsive elements. It meant a potential target gene might be found. Then we performed a dual Luciferase® Reporter analysis system (Promega, E1960, USA) according to its instruction. We choose DU145 cells and co-transfected them with plasmid-Lipo2000 (Invitrogen, USA) complexes, which including miR inhibitor, miR mimic or miR negative control (NC) as well as ERG fragment.

### Data analysis

All data were statistically analyzed using IBM SPSS Statistics 24.0 software package. The main statistical methods of this study included Student's t-test and one-way ANOVA. A statistical difference was considered when a p < 0.05.

## Results

### Negative regulation of miR-223-5p and ERG in different PCa cell lines

**Figure 1:**
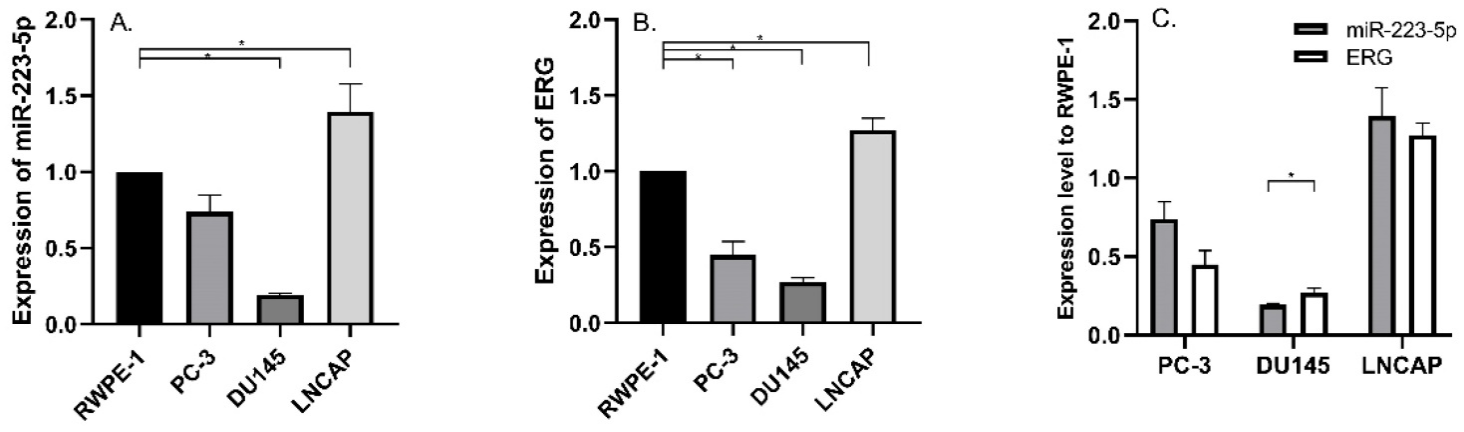
Aberrantly expressed miR-223-5p and ERG in different PCa cell lines. A: Compared to RWPE-1, miR-223-5p decreased in both PC-3 and DU145, while it was only up-regulated in LNCaP. B: Compared to RWPE-1, ERG significantly decreased in both PC-3 and DU145 cells, but it significantly increased in LNCaP. C: The expression level of ERG appeared to be negatively controlled by miR-223-5p in all three PCa cell lines. * represents p<0.05.

We detected miR-223-5p expression in three commonly used PCa cell lines and compared its expression in RWPE-1; and we found significant differences. Compared to RWPE-1, miR-223-5p was down-regulated in DU145 (p<0.05), while it was only up-regulated in LNCaP (p<0.05) (Figure 1A). Therefore, miR-223-5p was in the lowest level in DU145, while in LNCaP, its highest level was obtained.

Then we searched multiple target gene prediction websites (including Targetscan, miRDB, microRNA and PhastCons), in order to identify special target genes of miR-223-5p. The results from these three websites all suggested ERG might be one of them. Thus, ERG mRNA was detected in the above cells. Similar to miR-223-5p, we found ERG expression remarkably decreased in both PC-3 and DU145 (both p<0.05) than that in RWPE-1, but it was significant higher in LNCaP (p<0.05) (Figure 1B). In detail, ERG had the lowest expression level in DU145 and highest one in LNCaP, respectively. Combined with the above results of both miR-223-5p and ERG (shown in Figure 1C), we found that a negative correlation probably existed between the expression of ERG and miR-223-5p.Taken together, these above results, to some extent, we believed that miR-223-5p was likely to targeted inhibition of ERG mRNA expression even further confirmation might be needed. Thus, we selected DU145 cells for further following studies to confirm our hypothesis of miR-223-5p targeted inhibition of ERG.

### miR-223-5p negatively regulates the expression of ERG

**Figure 2:**
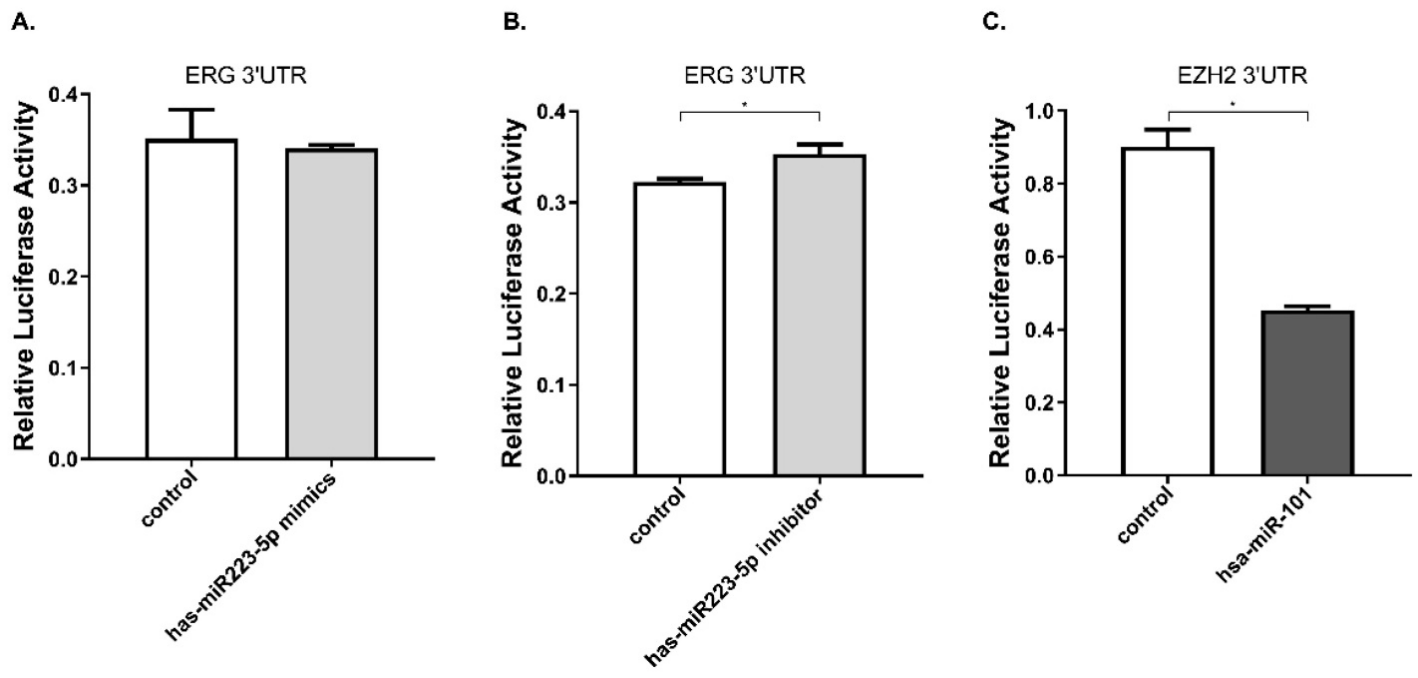
miR-223-5p specifically down-regulated ERG by targeting its 3' UTR. A. ERG was found a slightly decreased in cells with miR-mimics. B: Conversely, ERG presented a significantly increased in cells transfected by miR-inhibitor. C. has-miR-101 was set as a positive control to verify the validity. * represents p<0.05.

Furthermore, we performed luciferase reporter assay to verify whether miR-223-5p could specifically target and regulate ERG. The DU145 line was selected for this assay. The result confirmed this assumption. After cells transfected by mir-223-5p mimics (miR-MM), we observed ERG expression was slightly down-regulated, with a rate of about 97.2%; conversely, in DU145 cells with mir-223-5p antisense oligonucleotides (miR-AO) transfected, ERG expression was significantly increased with a 109.5% up-regulation rate (Figure 2A & B). has-miR-101 was set as a control to verify the validity (Figure 2C). At this point, it was reasonable to confirm that ERG could be negatively regulated by miR-223-5p and presented as a target gene.

### miR-223-3p promotes cell migration and invasion in PCa by targeting ERG gene

**Figure 3:**
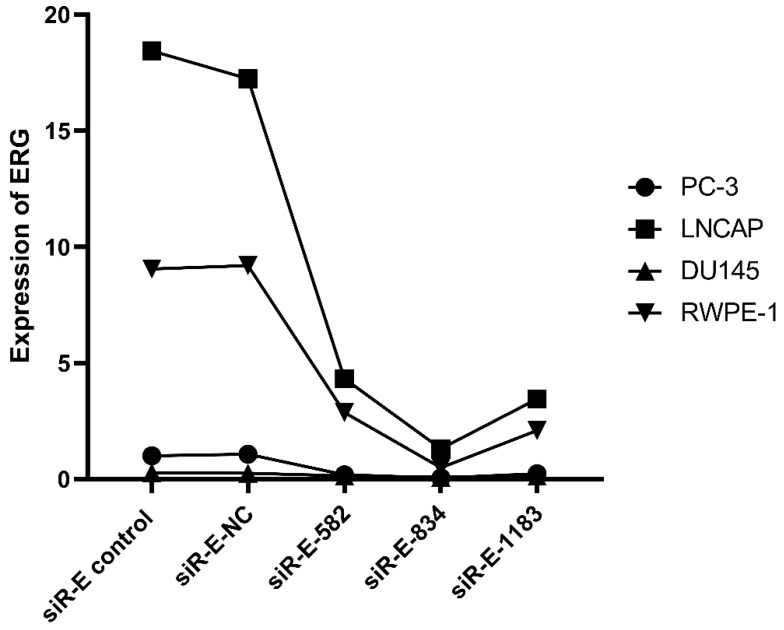
The level of ERG in different PCa cell lines after transfected with selected silence of ERG mRNA. Three types of silence mRNA were adopted, including siRNA-E-582, siRNA-E-834 and siRNA-E-1183. The RT-qPCR results confirmed that ERG mRNA significantly decreased in all PCa cells transfected with siRNA-ERG-834, obviously suggestive of siR-E-834 as the best gene-silencing vector. Scrambled siRNA as well as cells without any transfection were used as negative control (NC). or control for normalization.

We performed this study to further discovery ERG's role in the PCa oncogenesis through up or down regulating miR-223-3p expression. RWPE-1 and three PCa cell lines were cultured and then three ERG siRNAs were used to transfect these cells one by one in order to select the optimal ERG siRNA for subsequent studies, including siRNA-ERG-582 (Forward: 5'- GACGUCAACAUCUUGUUAUTT-3'; Revise: 5'-AUAACAAGAUGUUGACGUCTT-3', siR-E-582), siRNA-ERG-834 (Forward: 5'-CCUGAAGCUACGCAAAGAATT-3'; Revise: 5'-UUCUUUGCGUAGCUUCAGGTT-3', siR-E-834) and siRNA-ERG-1183 (Forward: 5'-GGAAGAGCAAACCCAACAUTT-3'; Revise: 5'-AUGUUGGGUUUGCUCUUCCTT-3', siR-E-1183). Scrambled siRNA as negative control (Forward: 5'-UUCUCCGAACGUGUCACGUTT-3'; Revise: 5'- ACGUGACACGUUCGGAGAATT -3', siR-E-NC). Additionally, cells without transfection were also used as a comparison. The RT-qPCR results confirmed that ERG mRNA was found excessively suppressed in cells transfected with siRNA-ERG-834. Thus, siRNA-ERG-834 was selected for the following study (Figure 3).

**Figure 4:**
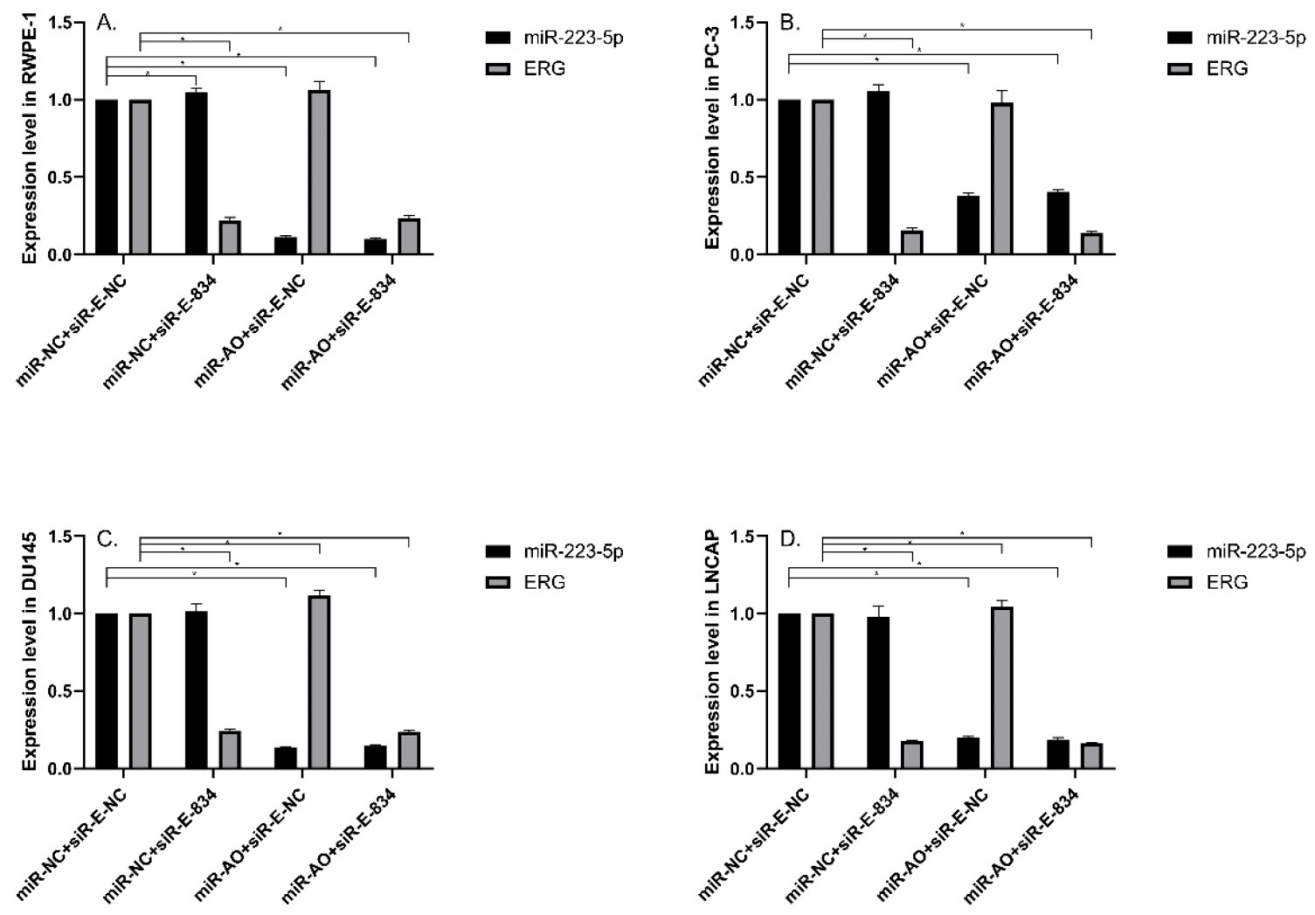
The level of miR-223-5p and ERG in different cells after transfected with miR-AO and/or siR-ERG-834. In all PCa cell lines, miR-223-5p in cells with co-transfection of miR-AO+siR-E-NC or miR-AO+siR-E-834 significantly decreased than that in cells with miR-NC+miR-E-NC or miR-NC+siR-E-834 (A-D) . In all PCa cell lines, the ERG mRNA expression was significantly lower in miR-NC +siR-E-834 and miR-AO+siR-E-834 groups than that miR-NC+siR-E-NC group (A-D). Conversely, ERG mRNA expression in each DU145 and LNCaP lines was significantly increased in miR-AO+siR-E-NC group when compared to miR-NC+siR-E-NC group (C & D). Additionally, miR-223-5p increased after cells transfected with siR-E-834 (A-C). * represents p<0.05.

**Figure 5:**
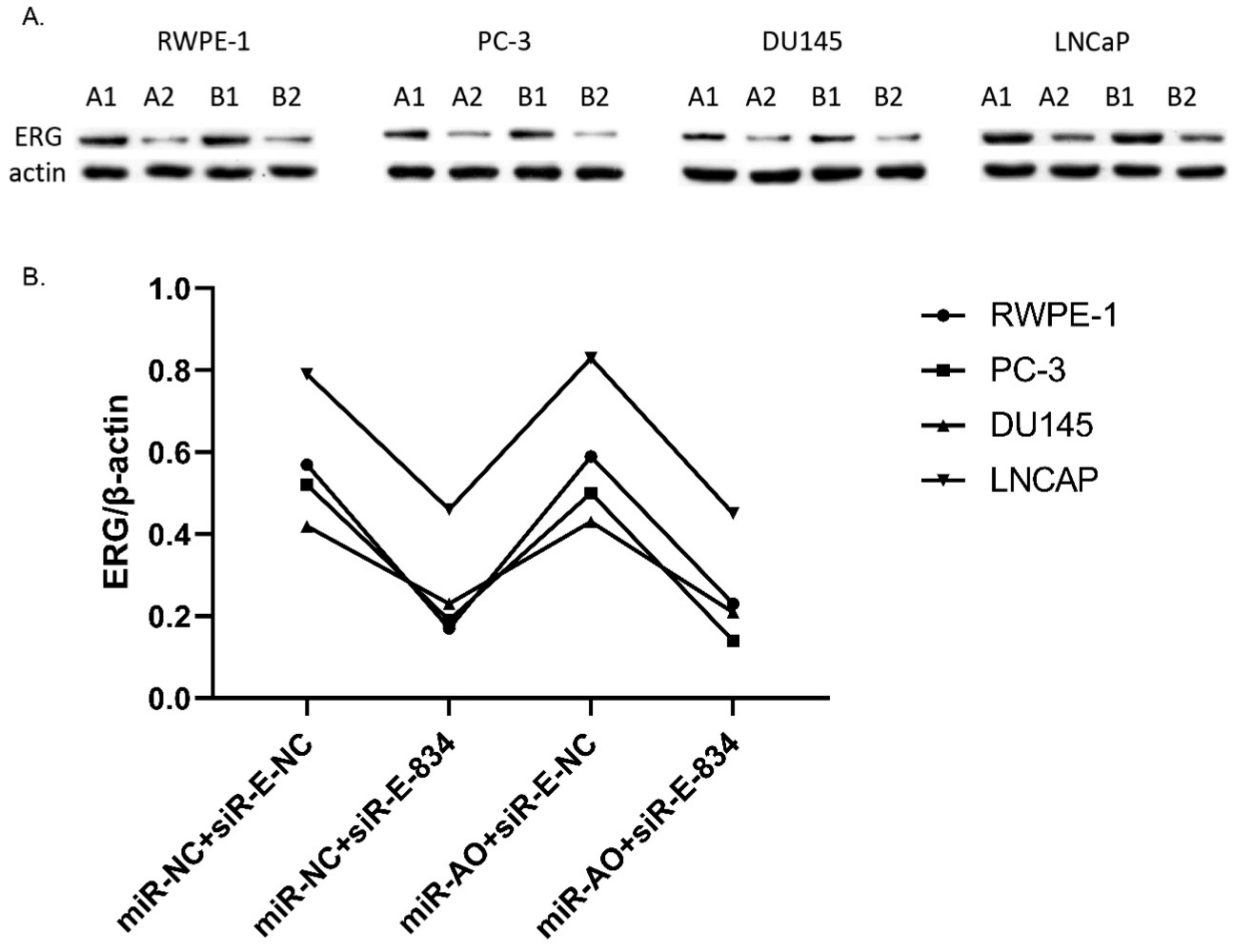
The level of ERG in cells after transfected with miR-AO and/or siR-ERG-834. As shown by A & B, ERG protein decreased in all cells transfected with miR-NC+siR-E-834 or miR-AO+siR-E-834 respectively, while increasing in both DU145 and LNCaP lines transfected with miR-AO+siR-E-NC. These results were consistent with ERG mRNA expression.

MiR-NC+siR-E-834 was used to co-transfected cells, in order to down-regulate ERG expression without affecting miR-223-5p. Similarly, to up-regulate ERG expression through suppressing miR-223-5p, miR-AO+siR-E-NC was co-transfected to cells. We used miR-NC+siR-E-NC co-transfected cells as controls. All the results were shown in Figure 4A-D. In all PCa cell lines, the miR-223-5p expression in cells with co-transfection of miR-AO + siR-E-NC or miR-AO+siR-E-834 were significantly decreased than that in the cells with miR-NC + miR-E-NC or miR-NC+siR-E-834. In all PCa cell lines, the ERG mRNA expression was significantly lower in cells with miR-NC +siR-E-834 or miR-AO+siR-E-834 than that in cells with miR-NC+siR-E-NC. Conversely, ERG mRNA expression in each DU145 and LNCaP lines was significantly increased in cells with miR-AO+siR-E-NC when compared to the cells with miR-NC+siR-E-NC. Additionally, as shown in cells with miR-NC+siR-E-834, miR-223-5p significantly increased after transfected siR-E-834 in RWPE-1 (p<0.05), suggestive of a feedback control loop between ERG and miR-223-5p. Moreover, ERG protein expression, consistent with its mRNA expression, was also decreased in all PCa lines transfect with miR-NC+siR-E-834 or miR-AO+siR-E-834 respectively, while increasing in both DU145 and LNCaP cells with miR-AO+siR-E-NC (Figure 5). Thus, the interference of expression was observed in both ERG mRNA and protein expression.

**Figure 6:**
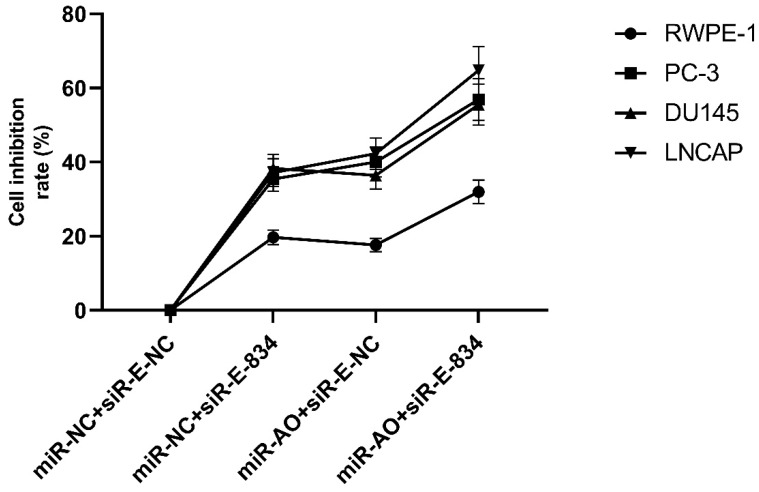
MTT assay of different PCa cell lines after transfected with miR-AO and/or siR-ERG-834. In cells with miR-NC+siR-E-834, when ERG mRNA and protein decreased, cells presented growth inhibition among four groups. The highest cell growth inhibition was seen in cells with miR-AO+siR-E-834.

**Figure 7:**
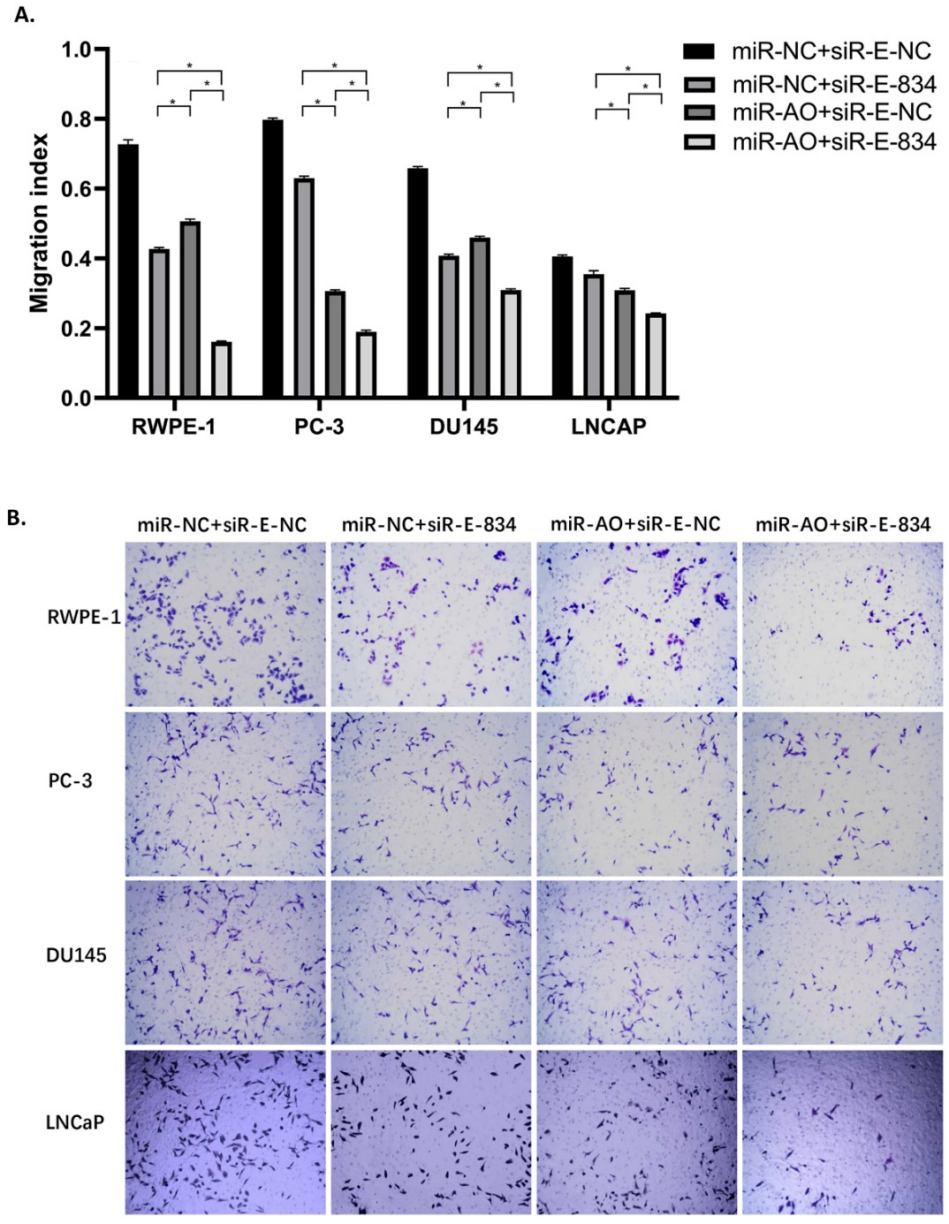
The migration assay of different PCa cell lines after transfected with miR-AO and/or siR-ERG-834. Consistent with MTT assay, in cells with miR-NC+siR-E-834, when ERG mRNA and protein decreased, cells presented lower abilities of migration among four groups. The lowest cell abilities of migration were seen in cells with miR-AO+siR-E-834. * represents p<0.05.

**Figure 8:**
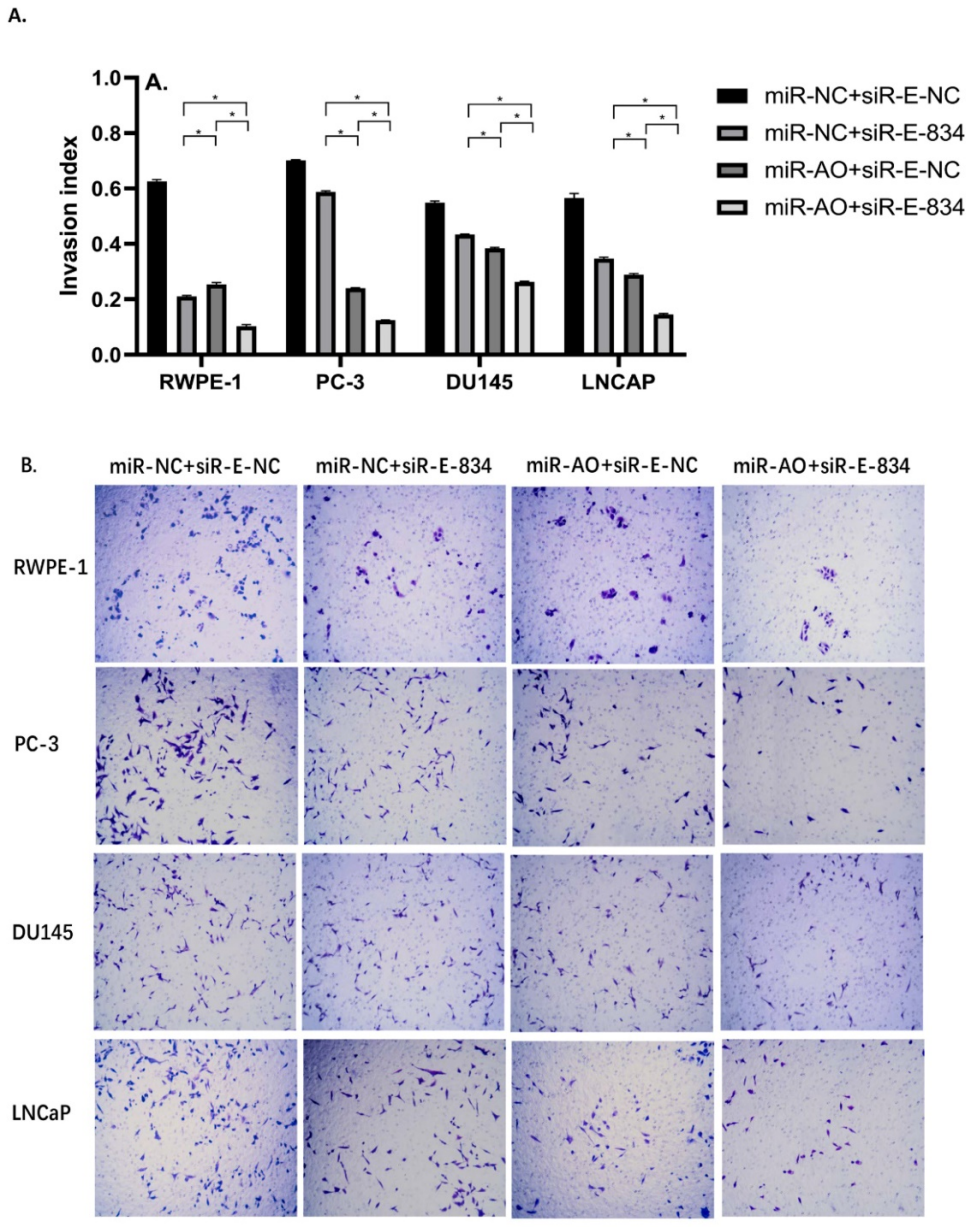
The invasion assay of different PCa cell lines after transfected with miR-AO and/or siR-ERG-834. Consistent with MTT assay and migration assay, in cells with miR-NC+siR-E-834, when ERG mRNA and protein decreased, cells presented lower abilities of invasion among four groups. The lowest cell abilities of invasion were seen in cells with miR-AO+siR-E-834. * represents p<0.05.

We further performed multiple assays to figure out the functions of ERG in PCa oncogenesis. ERG mRNA and protein decreased in cells with miR-NC+siRE-834, while cells presented higher rate of growth inhibition (Figure 6) and lower abilities of cell migration (Figure 7A & B) as well as cell invasion (Figure 8A & B) among the four cell groups with differently co-transfections. In cells with miR-AO+siR-E-834, we observed the highest cell growth inhibition and the lowest abilities of cell migration and invasion.

## Discussion

Accumulating studies suggest many effects of miR-223 in various cancers, mainly by influencing its target genes and interacting with multiple pathway networks. As an oncologic promotor or suppressor, it takes important part in extensive cancer cellular processes, including cell proliferation, and metastasis etc. 10-13. Particularly, miR-223 presents ectopic expression in different urogenital cancers such as bladder cancer 14, renal carcinoma 15 and testicular tumors 16. The current advances of its role in cancers have been summarized in a recent review 17. More deeply, miR-223-5p, has recently evidenced to be associated with cancer pathogenesis, including vulvar carcinoma 3, non-small cell lung cancer 4 and bladder cancer 5. However, the involvement of miR-223-5p in PCa remains obscure and worth of experimental investigation.

Generally, the lagging strand of miRNA derived from duplex miRNA is degraded without control of target gene expression 2, 18, 19. On the contrary, the guide strand of miRNA could form miRNA-Induced Silencing Complex to execute target gene silence. More recently, new findings have also indicated that some lagging strands of miRNAs could play a role in cancer pathogenesis. The examples of these types of strands include miR-144-5p 20, miR-145-3p 21, miR-149-3p 22, miR-150-3p 23 and so on.

Our present data showed that the passenger strand miR-223-5p decreased in two different cells (PC-3 and DU145) and possibly possessed anti-tumor functions, despite it can inhibit cancer cell proliferation; it can also negatively influence cell abilities of invasion as well as migration. These results were consistent with other groups' studies. For example, Sugawara et al found decreased miR-223-5p in bladder cancer tissues and by targeting ANLN gene, it could inhibit bladder cancer cells migration and invasion 5. Dou et al found in non-small cell lung cancer, decreased miR-223-5p was found in its tissues and cell lines, and could hold the anti-tumor roles in oncogenic progression through targeting E2F8^4^. Therefore, it is of necessity to discover potential targets of miR-223-5p and establish new strategies to inhibit its oncogenic signaling, which might contribute to new PCa treatment opinions.

**Figure 9:**
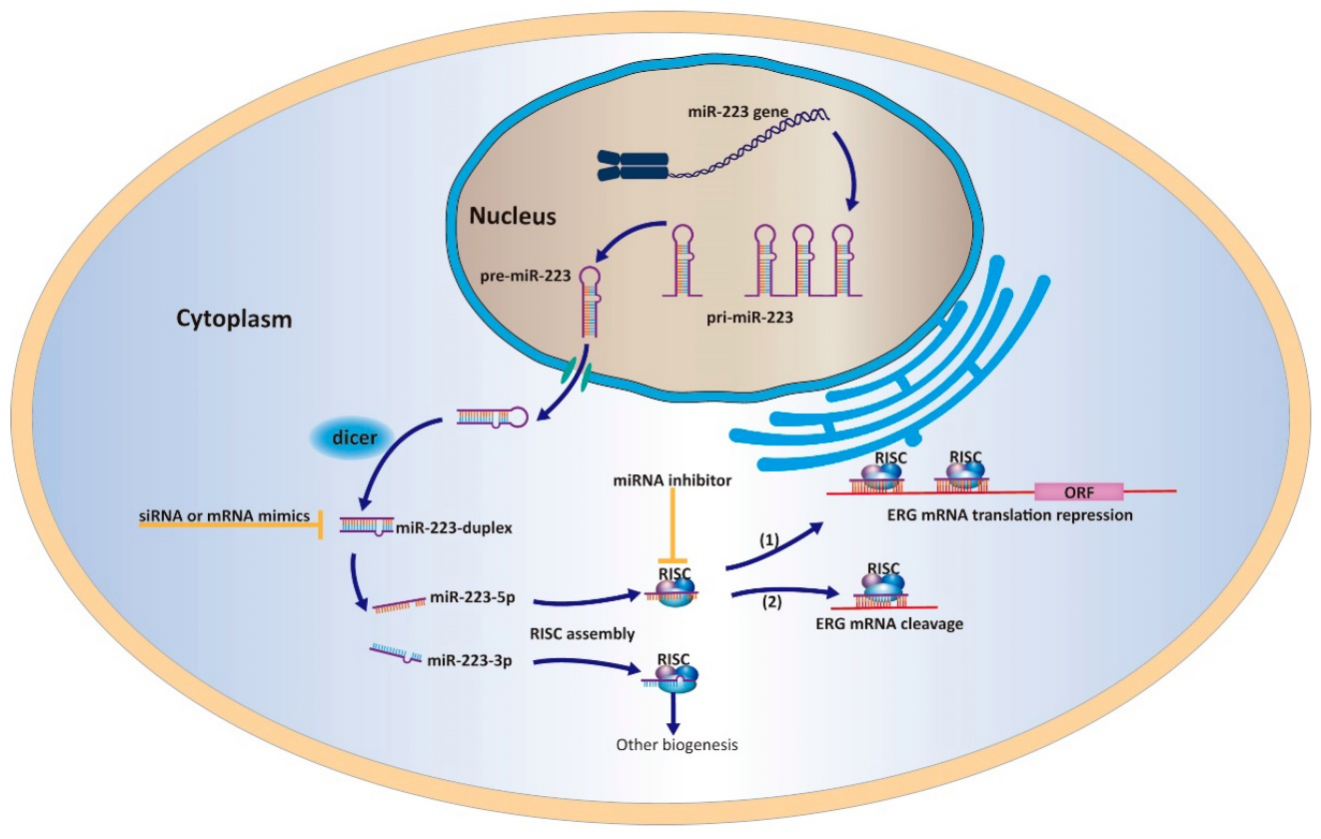
A work model elucidating that miR-223-5p could target ERG and inhibit its expression in PCa cells.

In this study, the molecular target gene regulated by miR-223-5p was analyzed to elucidate its involvement in PCa pathogenesis. Based on our previous strategy 9, we performed a miRNA target search by using several target gene prediction systems websites as mentioned above. Finally, ERG was chosen as a candidate for further study. ERG, discovered in 1987, is a member of the E-26 family. It is essential for the growth and development of a whole tissues and cells 6. In 2005, a paper by Deramaudt et al found TMPRSS2 and ERG could form into PCa-specific gene fusion, presenting as the androgen-driven promotor; and it is now confirmed as the most common form of gene fusions in PCa 24. After that, the study of TMPRSS2-ERG fusion becomes hot in PCa. Clinical studies have found it can be detected in one-third of African Americans and nearly half of Caucasians Americans as well as Asians 7, 25, 26. ERG is a famous oncogene as it is role in Ewing's sarcoma 27 and leukaemia 28. Also in PCa, ERG is considered to be an most consistent overexpressing oncogene, linking to clinical markers (high Gleason score, advanced tumor stage, metastasis etc.) and promotion of cell behaviors (proliferation, invasion and metastasis)^26^, ^29^. Our study was in line with the current observations of ERG. As shown by RT-qPCR results, ERG expression was increased in LNCaP cell line but decreased both PC-3 and DU145 cell lines. Despite this result is self-contradictory to a certain extent, we could still find that ERG was negatively regulated by miR-223-5p as shown in Figure 1. Subsequent Luciferase reporter assay also confirmed that ERG could be target-responsive controlled by miR-223-5p. Moreover, by silencing miR-223-5p showed a significantly increased expression of ERG in both DU145 and LNCaP cell lines, suggesting a possible feedback loop between ERG and miR-223-5p. After knockdown ERG by siR-ERG-834, all the PCa cells showed an obvious inhibition of cell proliferation, migration and invasion (Figure 6 & 7). Taken together, it is reasonable to speculate that ERG contributes to the carcinogenesis of PCa and its functions could be selectively reverse by miR-223-5p. The work model (Figure 9) elucidates the possible role of miR-223-5p in PCa. This might broaden the research scope and provide new opinion in PCa pathogenesis.

Particularly, our previous report determined the oncogenic function of the guide strand miR-223-3p in PCa and this continuing study characterized the tumor suppressive ability of passenger strand miR-223-5p. Combing two studies together, miR-223-duplex may exert a bidirectional function in the PCa pathogenesis through the cooperation between miR-223-3p and miR-223-5p. However, it remains unclear and needs further exploration whether the dominant strand of miR-223-duplex exists. The collaboration between the two strands of miR-223-duplex have found in other biological processes. For example, Qin et al have shown that miR-223-3p/-5p duplex had the potential role in cooperatively suppressing ischemic/reperfusion-induced cardiac necroptosis 30. In bladder cancer, double strands of miR-223-duplex possess tumor suppressor gene through influencing several onco-miRNA including ANLN 5. Similar type of miRNA duplex functioning with dual strands could be found such as miR-145-duplex and miR-150-duplex 21, 23. To be honest, our continuing studies firstly reported the two strands of miR-223-duplex could work together and affect PCa carcinogenesis.

Certain limitation should be addressed. The main one is that both miR-223-5 and ERG were significantly up-regulated in LNCaP cell line but down-regulated in PC-3 and DU145, leading to a conflict result. It is indeed hard to explain and might be partly arise from the different PCa properties. PC-3 cells were isolated from a patient with PCa metastasis to the vertebral body. DU145 cell line was derived from brain metastasis, while LNCaP was isolated orthotopically from prostate glands and regional lymph nodes. Both PC-3 and DU145 are thought to be androgen-independent but LNCaP is androgen-dependent 31. It means that LNCaP cell line could represent the early stage of PCa. As mentioned above, TMPRSS2-ERG fusion may stimulate multiple downstream signaling pathways and promote PCa early development 26.Therefore, it could be explained why ERG expression was at a higher level in LNCaP than other two PCa cell line in present study. Due to the existence of feedback loop between ERG and miR-223-5p, a higher expression of ERG may result in a higher expressed miR-223-5p. It quite meets our results as shown in Figure 1.

## Conclusions

In conclusion, we revealed tumor suppressive abilities of miR-223-5p in PCa by targeting ERG gene. It could serve as a fundamental supplement and extension of our previous study about miR-223-3p. To our knowledge, this is the first report about miR-223-5p and miR-223-3p might coordinately regulate cell biological behavior in PCa cells. Exploration of miR-233-duplex orientated pathway networks may help us develop novel potential therapeutic options for PCa.

## Figures and Tables

**Table 1:** Primers for quantitative polymerase chain reaction.

Gene	Primers (5′-3′)
Actin	F ACCCTGAAGTACCCCATCGAG
	R AGCACAGCCTGGATAGCAAC
ERG	F CGTGCCAGCAGATCCTACG
	R GGTGAGCCTCTGGAAGTCG
miR-223-5p	GCGCCCGTGTATTTGACAAGCTGAG
U6	F CTCGCTTCGGCAGCACA
	R AACGCTTCACGAATTTGCGT

Abbreviation: F, forward; R, reverse; ERG, the ETS-related gene.

## Abbreviations

ERG: ETS-related gene
miR-AO: mir-223-5p antisense oligonucleotides
miR-MM: mir-223-5p mimics
miRNA: MicroRNA
MTT: 3-(4,5-dimethylthiazol-2-yl)-2,5-diphenyl tetrazolium bromide
NC: negative control
PCa: prostate cancer
